# Three-Dimensional Model Analysis Revealed Differential Cytotoxic Effects of the NK-92 Cell Line and Primary NK Cells on Breast and Ovarian Carcinoma Cell Lines Mediated by Variations in Receptor–Ligand Interactions and Soluble Factor Profiles

**DOI:** 10.3390/biomedicines12102398

**Published:** 2024-10-20

**Authors:** Nadezhda A. Alekseeva, Anna A. Boyko, Marina A. Shevchenko, Maria V. Grechikhina, Maria A. Streltsova, Ludmila G. Alekseeva, Alexander M. Sapozhnikov, Sergey M. Deyev, Elena I. Kovalenko

**Affiliations:** Shemyakin & Ovchinnikov Institute of Bioorganic Chemistry, Russian Academy of Sciences, 117997 Moscow, Russia; nadalex@inbox.ru (N.A.A.); mshevch@gmail.com (M.A.S.); marygrec@mail.ru (M.V.G.); mstreltsova@mail.ru (M.A.S.); luda.alekseeva@mail.ru (L.G.A.); amsap@mail.ru (A.M.S.); biomem@mail.ru (S.M.D.)

**Keywords:** NK cells, NK-92, breast cancer, ovarian cancer, tumor spheroids model

## Abstract

**Background/objectives:** The functional activity of a certain tumor determines the effectiveness of primary NK cells and NK-92 cell line-based cancer therapy; their therapeutic effectiveness against different tumors can vary. This work provides a direct simultaneous comparison of the cytotoxic effects of in vitro-activated peripheral NK (pNK) cells and NK-92 cells in spheroid models of BT-474, MCF7 and SKOV-3 carcinomas and uncovers the reasons for the differential effectiveness of NK cells against tumors. **Methods:** Tumor spheroids of similar size and shape, obtained from agarose molds, were incubated with NK-92 or pNK cells for 24 h. Tumor cell death was detected using flow cytometry or confocal microscopy. Cytokine production, granzyme B levels and NK cell degranulation analyses were performed, along with pNK and target-cell phenotypic characterization. **Results:** While NK-92 and pNK cells lysed BT-474 spheroids with comparably low efficiency, pNK cells were more capable of eliminating MCF7 and SKOV-3 spheroids than NK-92 cells were. The results of the functional and phenotypic analyses strongly support the participation of the NKG2D-NKG2DL pathway in pNK cell activation induced by the most sensitive cytotoxic attack on SKOV-3 spheroids, whereas the CX3CR1-CX3CL1 axis appears to be involved in the pNK reaction against MCF-7 spheroids. **Conclusions:** We provide a new approach for the preliminary identification of the most promising NK cell receptors that can alter the effectiveness of cancer therapy depending on the specific tumor type. Using this approach, NK-92 cells or pNK subsets can be selected for further accumulation and/or genetic modification to improve specificity and reactivity.

## 1. Introduction

NK cells play an important role in antitumor immunity. They exert cytotoxic effects via a wide range of activating and inhibitory receptors and are capable of regulating adaptive immune responses through the production of cytokines and chemokines [1]. Due to their intrinsic ability to recognize and lyse tumor cells coupled with their low immunogenicity and relative safety, allogeneic NK cells may be advantageous over T cells in adoptive immunotherapy against cancer [2]. According to preclinical studies and early clinical trials, the use of NK cells as a component of adoptive cellular therapy has shown promising results in the arsenal of anticancer approaches [3].

The NK-cell-mediated destruction of tumor cells is based on the rapid activation of secretory activity and the release of cytotoxic molecules without prior sensitization. Many factors can influence the effectiveness of NK cell therapy, and these factors are primarily related to the functional activity against a specific tumor, access to tumor cells, the tumor microenvironment impact and the number of infused NK cells [4]. Immune surveillance of peripheral blood NK (pNK) cells and their infiltrative activity depend strongly on the individual characteristics of the donor. Alternatively, the NK-cell-like NK-92 cell line, which is characterized by activated peripheral NK cells and is more stable and reproducible than NK cells, was shown to be safe and to have minimal side effects in several clinical trials [5,6,7]. The NK-92 cells and pNK cells are phenotypically nonidentical. For example, NK-92 cells exhibit decreased expression of NKG2D, one of the most important activating receptors involved in tumor cell recognition and subsequent destruction [8,9]. Alternatively, specific antibodies against tumor antigens may trigger antibody-dependent cellular cytotoxicity (ADCC) in NK cells [10,11]. NK-92 cells are negative for CD16 and cannot mediate ADCC. However, the NK-92 cell line mostly lacks inhibitory killer-cell immunoglobulin-like receptors (KIRs), which might decrease the cytotoxicity of these cells [12]. However, studies examining the cytotoxic antitumor impact of pNK and NK-92 cells in various experimental designs, including gene-modified variants [13,14,15], studies exploiting direct in vitro comparisons of the antitumor effects of NK-92 cells and primary NK cells against the same target cells, are still limited [16,17,18,19,20]. Direct comparisons of the effects of NK-92 cells and pNK cells against specific tumor subtypes might provide a more comprehensive insight into which NK cells are more appropriate as effectors. These results may further lead to the selection of a strategy for genetic modification of the NK-92 cell line or primary donor NK cells to improve their therapeutic efficacy against specific breast or ovarian carcinomas.

In this work, the tumor-induced cytotoxic effects and secretory activity of pNK cells and the NK-92 cell line were simultaneously assessed and compared in models of spheroids derived from the BT-474, MCF7 or SKOV-3 cell lines. Human cancer cells of ovarian (SKOV-3) and mammary (MCF7, BT-474) origin are cell lines that are widely used and included in xenograft models because of their high tumorigenic potential [21,22,23]. Tumor spheroids represent an in vitro model system that allows us to obtain data with improved biological relevance [24] and may be considered a good model for screening new anticancer drug effects [25,26] and for evaluating the infiltration and cytotoxicity of immune cells, including NK cells [27]. An advantage of this approach is the maintenance of numerous intercellular signals that are lost when cells are grown in 2D format [28]. Our model maintains the uniformity of spheroids by allowing them to form inside agarose molds of similar size and shape [29], which enhances the reproducibility of the results. Individual tumor spheroids from each cell line were statistically analyzed as separate samples. Moreover, by simultaneously evaluating spheroids from three carcinoma cell lines, we were able to directly compare the tumor-dependent cytotoxic effects of pNK and NK-92 cells. Tumor spheroids from BT-474 cells showed similar low susceptibility to the pNK and NK-92 effectors. Moreover, NK-92 and pNK cells exerted cytotoxic effects on MCF-7 and SKOV-3 spheroids to different degrees. Mutual effector–target influences associated with the levels of both NK cell receptors and their ligands on tumor cells, along with the intensity of soluble factor production, contributed to the different cytotoxic impacts of NK-92 and pNK cells.

## 2. Materials and Methods

### 2.1. Tumor Cell Lines

BT-474, MCF-7, SKOV-3 and SKOV-Kat cells (a SKOV-3 cell line stably transfected with the far-red fluorescent protein Katushka; commercial name TurboFP635) [30]) were cultivated in DMEM (PanEco, Moscow, Russia) supplemented with 10% fetal calf serum (FCS; HyClone Labs, Logan, UT, USA) and 2 mM L-glutamine (PanEco). Prior to spheroid formation, an antibiotic–antimycotic solution (Sigma–Aldrich, St. Louis, MO, USA) was added to the cell suspension at a concentration of 2 mM.

### 2.2. Cultures of NK-92 Cells and Primary NK Cells

The NK-92 cell line was purchased from ATCC (Manassas, VA, USA). The NK-92-GFP cell line was generated by transducing NK-92 cells with viral particles containing the GFP reporter protein. The plasmid vector MIGR1 (Plasmid #27490) was used. Prior to addition to spheroids, these cell lines were cultivated in RPMI-1640 medium (PanEco) supplemented with 20% FCS (HyClone Labs), 2 mM L-glutamine (PanEco) and 2 mM sodium pyruvate (PanEco) and further supplemented with 200 U/mL interleukin (IL)-2 (Hoffmann La-Roche, Basel, Switzerland) and 5 γ/mL IL-15 (Sigma–Aldrich).

Peripheral blood NK cells (pNKs) were isolated by negative magnetic separation (Miltenyi Biotec, Bergisch Gladbach, Germany) from the peripheral blood mononuclear cells (PBMCs) of 9 healthy male and female donors, median age 30 years; the percentage of female and male donors was 45 and 55%, respectively (Supplementary Table S1). PBMCs were obtained by gradient centrifugation of blood samples in a standard Ficoll solution (PanEco). Informed consent for participation in the study was obtained from the local ethics committee of Pirogov Russian National Research Medical University from the donors prior to blood donation.

Freshly isolated pNK cells were activated and expanded in 24-well flat-bottom plates with feeder cells in an NK:Feeder = 2:1 ratio in NK MACS cell medium (Miltenyi Biotec) supplemented with 10% FCS (HyClone Labs), 10% 2 mM L-glutamine, 2 mM sodium pyruvate (PanEco), 2 mM antibiotic-antimycotic solution (Sigma–Aldrich) and 100 U/mL recombinant IL-2 (Sigma–Aldrich) for 7 days. The culture medium was replaced every 3–4 days. As feeder cells, K562 cells genetically modified with membrane-bound IL-21 (K562-mbIL21) [31] were used. Before being added to NK cells, K562-mbIL21 cells were γ-irradiated at 100 Gy and stored in FCS (HyClone Labs) supplemented with 10% DMSO (Sigma–Aldrich) at −135 or −150 °C.

### 2.3. Models of Cellular Spheroids

Spheroids were formed in 9-well gelled agarose plates prepared using 3D-printed molds as previously described [29]. The spheroids were obtained by adding BT-474, MCF-7, SKOV-Kat or SKOV-3 cell suspensions in RPMI-1640 medium (PanEco) supplemented with 10% FCS (HyClone Labs) to wells (20,000 cells per well) of agarose gel (PanEco) plates placed into a standard 12-well plastic plate for culturing (Thermo Fisher Scientific, Waltham, MA, USA). The seeded cells were incubated for six days in a CO_2_ incubator (HyClone Labs) at 37 °C and 5% CO_2_; half of the RPMI-1640 medium was replaced on day 3. The self-assembled spheroids were visualized before further analysis using an Axio Lab A1 stereomicroscope (Carl Zeiss, Germany). A 4x objective was used to image spheroids in the wells of the plate. Mixed cultures of effector pNK or NK-92 cells with target spheroids at an E:T ratio = 3:1 were incubated for 24 h at 37 °C and 5% CO_2_ in the presence of 500 U/mL IL-2.

### 2.4. Spheroid Staining

Assessment of spheroid viability and NK cell cytotoxicity and phenotyping was performed by flow cytometry and confocal microscopy. For the confocal-microscopy-based viability assay, SYTOX Blue Dead Cell Stain (SytoxBlue) (Invitrogen, San Jose, CA, USA) was added to the whole intact spheroids for 1 h. Prior to the flow-based analysis, the spheroids with NK cells were washed with PBS (PanEco), treated with Accutase (Sigma–Aldrich) for 15 min, vigorously dispersed by pipetting and washed in PBS. NK cells were stained externally with the following mouse anti-human antibodies (Abs): KIR2DL2/3-FITC (clone REA1006), CD16-FITC (clone REA423), CD107a-APC (clone REA792), CD56-APC-Vio770 (clone REA196) (Miltenyi Biotec), TIM-3-BV-421 (clone F38-2E2), CX3CR1-APC (clone 2A9-1), galectin-9-PE (clone 9M1-3) (BioLegend, San Diego, CA, USA) and CEACAM-FITC (clone 117) (Sino Biological, Beijing, China). Indirect staining of NKG2D ligands with anti-hMICA/B (clone 159207), anti-hULBP1 (clone 170818), anti-hULBP2/5/6 (clone 165903), anti-hULBP3 (clone 166510) (R&D Systems, Minneapolis, MN, USA) Abs and secondary goat anti-mouse IgG-AF-647 (clone A21235, Molecular Probes) was performed. The levels of granzyme B (GrB) and IFNγ were assessed using an intracellular staining protocol (Inside Stain Kit, Miltenyi Biotec) with anti-human granzyme B AF-647-conjugated (clone GB11, BioLegend) and anti-IFNγ-FITC (clone B27, SonyBiotechnology, San Jose, CA, USA) antibodies, respectively.

The Ab-stained cells were incubated for 30 min at 4 °C in PBA staining buffer (PBS supplemented with 0.5% BSA (Serva, Heidelberg, Germany) and 0.01% sodium azide (AMRESCO, Inc., Aurora, CO, USA)) and then washed with PBS. Immediately before flow acquisition, the cells were stained with SytoxBlue viability dye (Invitrogen).

### 2.5. Flow Cytometry

For acquisition of flow cytometry data, a MACSQuant 10 cytometer (Miltenyi Biotec) equipped with 405 nm, 488 nm and 635 nm lasers was used. No fewer than 15,000 events were recorded for SKOV-3, BT-474 and MCF7 cells in an FSC/SSC gate, and no fewer than 25,000 events were recorded for the pNK/NK-92 cell fractions.

### 2.6. Degranulation Assay

Analysis of the natural cytotoxic activity of NK cells was conducted via flow cytometry by assessing degranulation in response to incubation with spheroids. The level of degranulation corresponding to the lysosomal marker LAMP-1 (CD107a) on the surface of NK cells was determined using a CD107a monoclonal antibody. NK cells were restimulated overnight with 500 U/mL IL-2 (Sci-Store, Moscow, Russia) and added to spheroids in agarose wells in DMEM (PanEco) supplemented with the CD107a antibody and 10 μg/mL brefeldin A (Sigma-Aldrich) to prevent fluorescence inactivation due to the reuptake of vesicles containing the LAMP-1 molecules. Then, the cells were incubated for 24 h at 37 °C and 5% CO_2_. For NK cell phenotype assessment, a mixture of antibodies (against CD56, CD57, KIR2DL2/3, CD16 and NKG2C) was used. NK cells were identified as CD56^+^ cells. Analysis was performed on a MACSQuant 10 flow cytometer (Miltenyi Biotec); the percentage of CD107a^+^ NK cells was evaluated.

### 2.7. Multiplex Analysis of Soluble Analytes

The levels of cytokines (GM-CSF, Granzyme B, IFNγ, IL-6, MCP-1 (CCL2) and TNF-α) in the supernatants of control samples containing pNK, NK92 or tumor spheroid (MCF7, BT-474 or SKOV-3 cell lines) alone or in mixed samples of NK or NK-92 cells with spheroids were assessed via a flow-cytometry-based method using a MacsPlex Cytotoxic T/NK Cell Kit (Miltenyi Biotec). On days 5–6, each spheroid was transferred from the agarose mold to a 96-well plate (9 spheroids per well), after which 0.54 × 106 effector cells (NK or NK92) were added to a volume of up to 200 µL/well for 24 h at 37 °C and 5% CO_2_. After incubation, the samples were mixed and sedimented and the supernatants were collected and frozen for further analysis.

### 2.8. Confocal Microscopy and Quantitative Image Analysis

Three-dimensional images were obtained using a Zeiss LSM980 inverted confocal laser scanning microscope (Carl Zeiss, Oberkochen, Germany). A 10x objective (NA = 0.3) was used to image the spheroids. To image dead nuclei (SytoxBlue) and live SKOV-kat cells, 445 and 594 nm lasers, respectively, were used. Emission was acquired in λ-mode using a 32 (+2)-channel QUASAR detector (Carl Zeiss) set to a 405–695 nm range with the following spectral unmixing using ZEN 2012 SP5 software (Carl Zeiss).

The images were acquired as Z-stacks and subjected to processing using Imaris version 9.8 software (Oxford Instruments, Abingdon, Oxfordshire, UK). For quantitative analysis, 200 µm sections were obtained from each spheroid. Cells and nuclei were processed as spots based on the maximum intensity projections. The estimated diameter of a single spot was 7 µm for NK-92-GFP nuclei, 10 µm for SKOV-kat nuclei and 25 µm for SKOV-kat cells. The number of spots was quantified using the Imaris statistic option.

The final images were processed using Adobe Photoshop CS version 5 (Adobe Systems, Mountain View, CA, USA).

### 2.9. Statistical Analysis

Flow cytometry data were analyzed using FlowJo X 10.0.7r2 (FlowJo LLC, Ashland, OR, USA) and GraphPad Prism 8.00 ver. software (StatSoft, Inc., Tulsa, OK, USA). For normally distributed data, a *t* test or one-way ANOVA was performed. For nonnormally distributed data, the Mann–Whitney U test or multiple comparison Kruskal–Wallis test was used. The results are shown as the mean ± SD unless otherwise indicated. A value of *p* < 0.05 was considered to indicate statistical significance. * *p* < 0.05, ** *p* < 0.01, *** *p* < 0.005, **** *p* < 0.0001.

## 3. Results

### 3.1. NK-92 and Peripheral NK Cells Exhibit Different Cytotoxic Effects Depending on the Kind of Tumor Spheroid Cells

To assess the anticancer cytotoxic effects of peripheral NK (pNK) and NK-92 cells, 3D-culture tumor cell models were established. By day 6, the SKOV-3, BT-474 and MCF7 cells had formed dense agarose mold cell clusters of similar shape (Supplementary Figure S1), which were subsequently analyzed as separate spheroid samples. The effects of NK-92/pNK cells on spheroid cell viability were measured in the experimental samples (one spheroid + NK-92/pNK cells) after 24 h of incubation in the presence of 500 U/mL IL-2. Prior to flow cytometry analysis, spheroids were dissociated using Accutase, and SytoxBlue viability dye was applied to detect dead cells in the cell suspension. The proportions of nonviable (SytoxBlue^+^) cells were compared between intact and NK-cell-treated spheroid samples, and the results are presented as upregulated (>1) or downregulated (<1) fold changes. To distinguish NK cells by fluorescence, pNK and NK-92 cells were stained with an anti-CD56 antibody, and the NK-92 cells were additionally GFP-modified.

**Figure 1:**
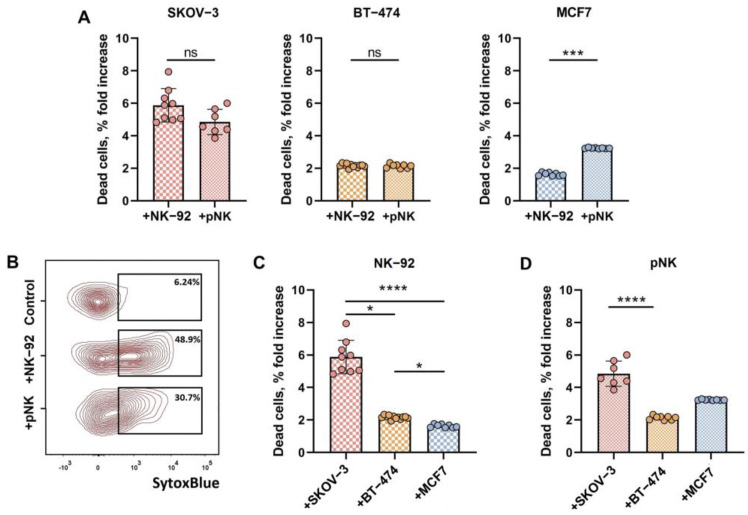
Reduced viability of SKOV-3, BT-474 and MCF7 cells in spheroid models after coculture with NK-92 or pNK cells, measured by flow cytometry. (**A**) Proportions of dead (SytoxBlue^+^) cells in the spheroids of SKOV-3, BT-474 and MCF7 cells after coincubation with NK-92 cells compared to those with the pNK effectors. (**B**) Proportions of dead (SytoxBlue^+^) cells in the spheroids of SKOV-3 incubated with NK-92 or pNK cells. Representative flow cytometry data. (**C**) Comparison of the proportions of dead (SytoxBlue^+^) cells in the spheroids of the SKOV-3, BT-474 and MCF7 cell lines after coincubation with the NK-92 effectors. (**D**) Comparison of the proportions of dead (SytoxBlue^+^) cells in the spheroids of SKOV-3, BT-474 and MCF7 cells after coincubation with cells in pNK effector groups. E:T = 3:1 in the presence of 500 U/mL IL-2, incubation time t = 24 h. Statistical analyses were performed using a Mann–Whitney U test (**A**) or a Kruskal–Wallis multiple comparison test (**B**,**C**) (ns—non significant,* *p* < 0.05, *** *p* < 0.005, **** *p* < 0.0001); means ± SDs are shown.

All three types of tumor-cell spheroids showed susceptibility to the cytolytic action of both NK-92 cells and pNK cells, although to varying degrees. Compared with those from BT-474 and MCF7 breast cancer cells, the spheroids from SKOV-3 ovarian carcinoma cells were less resistant to NK cell attack, and both pNK cells and NK-92 cells exhibited comparably low abilities to eliminate BT-474 cells (Figure 1). Flow cytometry analysis did not reveal any difference between the ability of NK-92 and pNK cells to induce cell death in SKOV-3 and BT-474 spheroids, whereas MCF7 spheroids were more susceptible to pNK cytotoxic attack under the same experimental conditions than were NK-92 effector cells (Figure 1A,B). Among the tumor cell lines tested, the lowest susceptibility to NK-92 cell action was shown for the MCF7 spheroids (Figure 1C), whereas after pNK addition, a significantly lower proportion of dead cells was observed for the BT-474 spheroids than for the SKOV-3 cells (Figure 1D).

Flow-cytometry-based single-cell measurements require dissociation of the spheroids, while microscopy-based assays provide the ability to measure the parameters of an intact spheroid located directly in the same agarose well where it grew and evaluate target cell loss in the spheroid. Since SKOV-3 spheroids appeared to be the most sensitive to the cytotoxic impact of NK cells, NK-92- and pNK-mediated cytotoxicity was assessed via confocal laser scanning microscopy (CLSM) in the 3D model of these cells. The CLSM method is restricted by the use of fluorescently labeled target tumor cells; therefore, previously obtained SKOV-3 cells stably expressing the fluorescent protein Katushka (SKOV-Kat) [29] were used for spheroid formation. Preliminary flow cytometry analysis showed comparable NK-92- and pNK-cell-mediated cytotoxic effects on intact SKOV-3 and SKOV-Kat spheroids (Supplementary Figure S2A,B).

**Figure 2:**
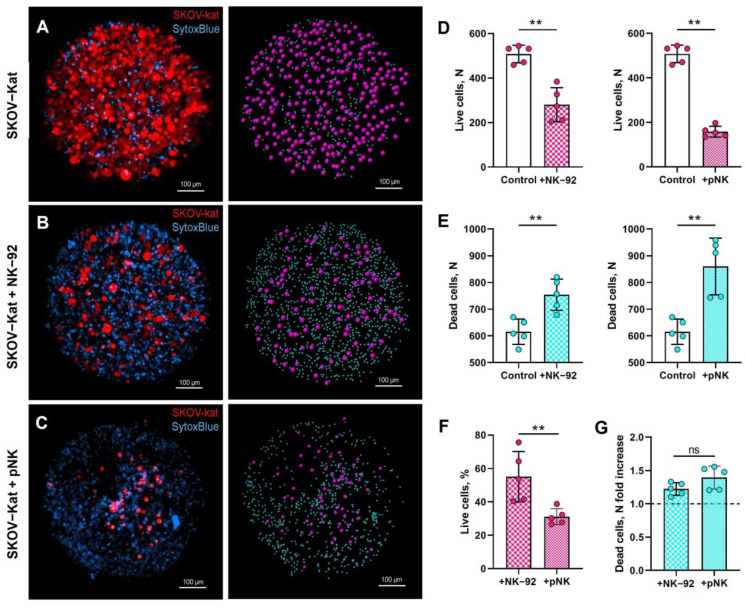
CLSM-based evaluation of the cytotoxic effects of NK-92-GFP and pNK cells on a SKOV-Kat-cell spheroid model. (**A**–**C**) Representative images of a SKOV-Kat-derived control spheroid (**A**), a SKOV-Kat spheroid incubated in the presence of NK-92-GFP cells (**B**) and pNK cells (**C**) at a ratio of E:T = 3:1. SKOV-Kat cells (red) and SytoxBlue-positive nuclei (blue) are represented via volume rendering (**left**) or via spots (SKOV-Kat, magenta; SytoxBlue, cyan) (**right**). The scale bar is 100 µm. The numbers of live cells (Katushka^+^) (**D**) and dead cells (SytoxBlue^+^) (**E**) were measured in spheroids incubated with (SKOV-Kat + NK-92, SKOV-Kat + pNK) or without effectors (SKOV-Kat). (**F**) Live cell (Katushka^+^) proportions in spheroids incubated with NK-92 cells and pNK cells. (**G**) Dead cell fold increase in the spheroids incubated with NK-92 and pNK cells. Statistical analysis was performed using a Mann–Whitney U test (ns—non significant, ** *p* < 0.01); the means ± SDs are shown.

The experimental design included the use of CD56-labeled preactivated pNK cells or NK-92-GFP cells coincubated for 24 h with the individual SKOV-3-Kat spheroids in agarose wells. CLSM was used for 3D cell viability analysis. SytoxBlue dye was added immediately before the measurements were taken. Quantitative analysis of Katushka-expressing cells in spheroids was performed by constructing SKOV-Kat surfaces in the selected fragment. A decrease in the proportion of viable cells in the region of the red spectrum (corresponding to Katushka fluorescence) and a simultaneous increase in the number of SytoxBlue^+^ cells in spheroids after NK-92 (Figure 2B,D,E) and pNK (Figure 2C–E) coculture compared to those in control samples (intact spheroids) were shown. The efficiency of the tumor cell killing capacity, as assessed by the Katushka^+^ cell numbers, was greater for pNK cells than for NK-92 cells (Figure 2F). A similar trend toward an increase in the cytotoxic effect of pNK cells against SKOV-Kat spheroids compared to that of NK-92 cells was shown by SytoxBlue^+^ cell proportion measurements (Figure 2G), which corresponded to the results of the flow cytometry evaluation of tumor cell viability. Thus, the use of CLSM imaging of 3D cell cultures allowed us to detect the superior cytotoxic effect of pNK cells on SKOV-3-derived spheroids. Moreover, the strong correlation between the number of live Katushka^+^ cells and the number of dead SytoxBlue^+^ cells (Supplementary Figure S2C) confirmed the applicability of the viability-dye-based assay for both fluorescence microscopy and flow cytometry.

### 3.2. SKOV-3-Derived Spheroids Promote IFNγ, TNF and GM-CSF Secretion by Peripheral Blood NK Cells

**Figure 3:**
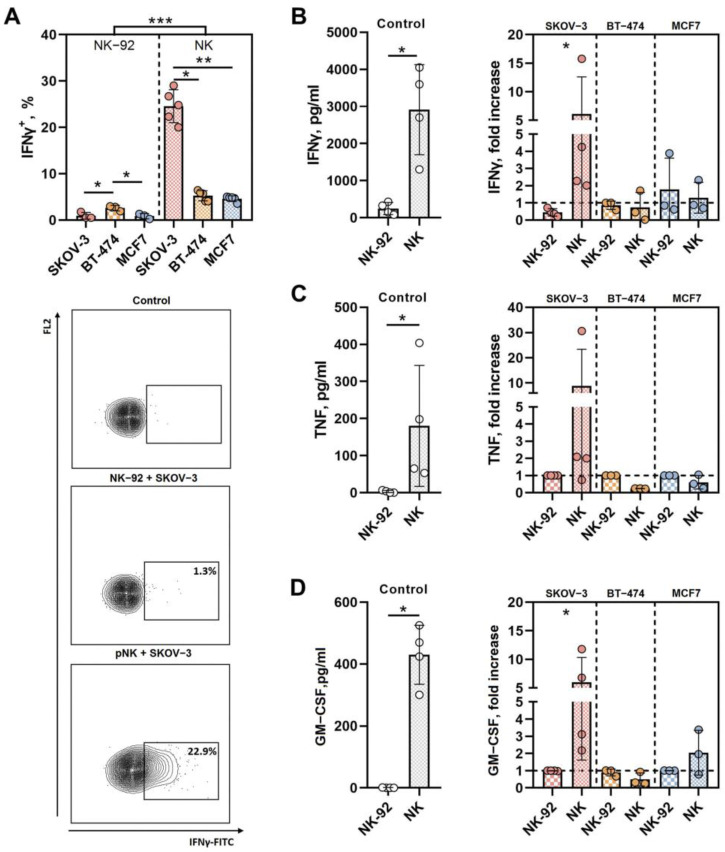
Differences in the production of cytokines by NK-92 and pNK cells in response to SKOV-3, BT-474 and MCF7 spheroids. (**A**) Proportions of IFNγ-positive NK-92 and pNK cells in culture after incubation with SKOV-3, BT-474 and MCF7 spheroids. (**B**) IFNγ levels in culture supernatants of NK-92 or pNK cells without spheroids (**left**) or with SKOV-3, BT-474 and MCF7 spheroids (**right**). (**C**) TNF levels in culture supernatants of NK-92 or pNK cells without spheroids (**left**) or with SKOV-3, BT-474 and MCF7 spheroids (**right**). (**D**) GM-CSF levels in culture supernatants of NK-92 or pNK cells without spheroids (**left**) or with SKOV-3, BT-474 and MCF7 spheroids (**right**). E:T = 3:1, incubation time t = 24 h. Statistical analysis was performed using a Mann–Whitney U test or a Kruskal–Wallis multiple comparison test (* *p* < 0.05, ** *p* < 0.01, *** *p* < 0.005); the means ± SDs are shown.

The natural cytotoxicity of pNK cells is often accompanied by an increase in the production of specific immune-active cytokines, usually proinflammatory cytokines. These cytokines play a regulatory role and can boost the activity of immune cells in the tumor milieu. In particular, the phenotype of activated NK cells is characterized by IFNγ, TNF, and GM-CSF upregulation. We analyzed the intracellular IFNγ accumulation in NK-92 and pNK cells in the presence of SKOV-3, BT-474 and MCF7 spheroids and secretion of IFNγ, TNF-α and GM-CSF into the extracellular space by NK-92 and pNK cells in control samples (in the presence of IL-2 only) or in response to the spheroid addition. The proportion of cells producing IFNγ in response to incubation with spheroids from all three analyzed lines was significantly lower among the NK-92 effector cells than among the pNK cells. The highest percentage of IFNγ-positive NK cells was observed in samples from SKOV-3 spheroids (Figure 3A). Similarly, the IFNγ concentration was also greater in the pNK cell culture supernatants than in the NK-92 cell culture supernatants, and an increase in the IFNγ level in response to the addition of spheroids to the pNK cells (fold change) was recorded only in the samples from the SKOV-3 cells (Figure 3B).

The concentration of TNF in control samples of pNK cells exceeded the level of this cytokine in NK-92 samples; the background level of TNF production by NK-92 cells was low and did not increase in the presence of spheroids (Figure 3C). An increase in TNF secretion by NK cells in response to the SKOV-3 target spheroids was shown for some donors, and a trend toward a decrease in TNF level was observed after incubation with the BT-474 and MCF7 spheroids.

In our model, NK-92 cells did not produce GM-CSF, although this factor was detected in control samples containing pNK cells (Figure 3D). Like that of TNF, the secretion of GM-CSF by pNK cells steadily increased, although to varying degrees, when the cells were cocultivated with SKOV-3 spheroids. For some donors, the accumulation of GM-CSF in pNK cell supernatants was also induced by the addition of MCF7 spheroids (Figure 3D).

Thus, analysis of the proinflammatory cytokine profile showed that pNK cells were more secretory than NK-92 cells in this model. SKOV-3 spheroids were again highlighted as more effective tumor targets for pNK cell activation than were BT-474 or MCF-7 spheroids.

### 3.3. Various Degrees of NK-Cell-Mediated Cytotoxicity Against SKOV-3, BT-474 and MCF7 Spheroids Are Associated with Different Patterns of NKG2D, Tim-3 and CX3CR1 Surface Expression in pNK Cells

The NK cell response to malignant cells includes the accumulation of lytic granules followed by degranulation. The phenotypic diversity among pNK cells suggested that degranulation toward different target cells may have several features that determine the cytotoxicity outcome. The surface expression of LAMP1/CD107 is a general marker of pNK cell cytolytic activity [31]. We assessed the proportion of CD107a^+^ pNK cells after coincubation with the tumor targets in the 3D model and analyzed the expression levels of several important NK cell markers and receptors associated with the cytotoxic response within this fraction.

**Figure 4:**
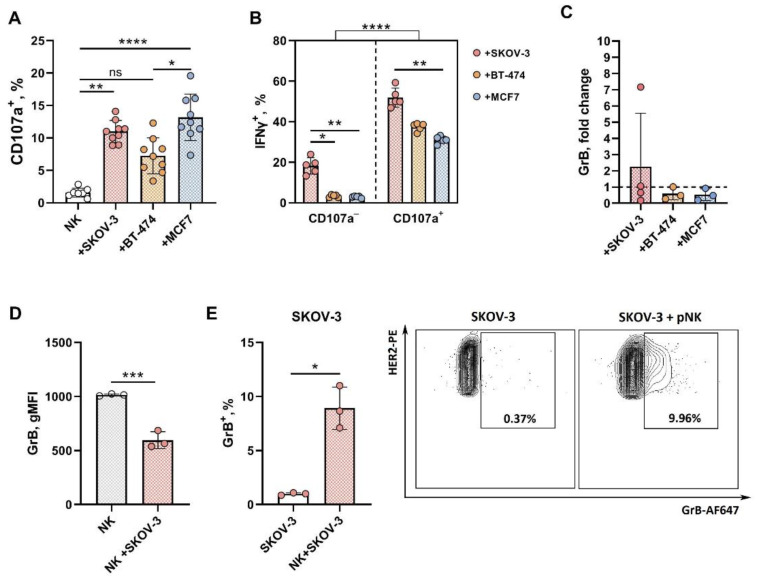
Cytotoxicity-associated factors in pNK cells in response to the SKOV-3, BT-474 and MCF-7 spheroids. (**A**) Degranulation activity and (**B**) intracellular IFNγ content in resting (CD107a^−^) and degranulated (CD107a^+^) pNK cells. (**C**) Granzyme B (GrB) accumulation in supernatants after coincubation of pNK cells with tumor spheroids; (**D**) Intracellular level of GrB in pNK cells (CD56^+^) alone and after interaction with SKOV-3 spheroids; (**E**) Intracellular level of GrB in SKOV-3 cells (HER2^+^, dissociated from spheroids) alone and after interaction with pNK cells. E:T = 3:1, incubation time = 24 h. Statistical analysis was performed using a Mann–Whitney U test or a Kruskal–Wallis multiple comparison test (ns—non significant, * *p* < 0.05, ** *p* < 0.01, *** *p* < 0.005, **** *p* < 0.0001); the means ± SDs are shown.

In accordance with the cell viability results, pNK cell degranulation depended on the type of cells forming the spheroids. We observed greater numbers of CD107a^+^ pNK cells after coincubation with SKOV-3- and MCF-7-derived spheroids than after coincubation with control cells (Figure 4A). As expected, the proportion of IFNγ-producing cells among degranulated CD107a^+^ pNK cells exceeded the IFNγ production level in the CD107a-negative pNK cell fraction for all three types of tumor spheroids analyzed (Figure 4B). Moreover, despite the comparable levels of degranulation in response to SKOV-3 and MCF7 cells, among the pNKs degranulating during incubation with SKOV-3 spheroids, a greater proportion of IFNγ-producing cells was observed than was observed for the pNK s, which degranulated in the presence of MCF7 cells (Figure 4A,B).

Directed release of lytic molecules into target cells is the primary mechanism of NK-cell-mediated cytotoxicity. With 3D models of NK/tumor cell interactions, we assessed the extracellular and intracellular levels of granzyme B (GrB), which is one of the major components of lytic granules closely related to the degranulation process. The levels of soluble GrB did not differ between the supernatants of control pNK and NK-92 cell samples, and neither type of tumor spheroid induced extracellular accumulation of this protein (Supplementary Figure S3A, Figure 4C). Instead, a slight decrease in the intracellular GrB level was recorded in pNK cells after 24 h of coincubation with the SKOV-3 spheroids, which were shown to be the most sensitive to NK cells in our model (Figure 4D), whereas approximately 10% of the SKOV-3 cells in the mixed culture became GrB positive (Figure 4E).

The newest NK cell classification defines three main NK cell clusters. NK1 cells have pivotal cytotoxic effector functions and are characterized by high expression of CD16 and CX3CR1, inhibitory KIRs and T-cell immunoglobulin and mucin-containing domain (Tim-3) [32]. The NK2 cluster, which comprises less differentiated NK cells, is characterized by a high density of NKG2D, one of the main natural cytotoxicity receptors.

**Figure 5:**
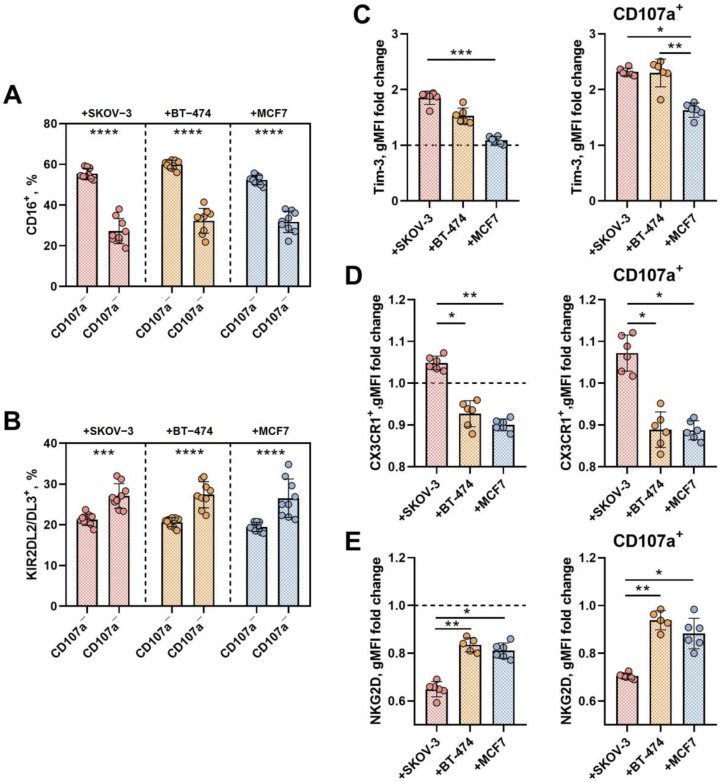
Analysis of surface markers related to cytotoxic activity and differentiation stages in total pNK cells and in the fraction of degranulated CD107a^+^ and resting CD107a^−^pNK cells after coincubation with tumor spheroids consisting of BT-474, MCF-7 or SKOV-3 cells. (**A**,**B**) Comparison of the proportions of degranulated and nondegranulating NK cells expressing CD16 (**A**) or KIR2DL2/3 (**B**). (**C**) The fold increase in the Tim-3 expression level (gMFI) in pNK cells after coincubation with SKOV-3-, BT-474- and MCF7-derived spheroids compared to that in pNK cells incubated without target cells. The fold change in Tim-3 gMFI in the total pool of pNK cells incubated with spheroids (left) and the fold change in Tim-3 gMFI in the population of degranulated CD107a^+^ pNK cells (**right**) are shown. (**D**) The CX3CR1 expression level (gMFI) in CX3CR1^+^ pNK cells after coincubation with SKOV-3-, BT-474- and MCF7-derived spheroids compared to that in pNK cells incubated without target cells. CX3CR1 gMFI in the total pool of pNK cells incubated with spheroids (**left**) and CX3CR1 gMFI in the population of degranulated CD107a^+^ pNK cells (right). (**E**) The fold increase in the NKG2D expression level (gMFI) in pNK cells after coincubation with SKOV-3-, BT-474- and MCF7-derived spheroids compared to that in pNK cells incubated without target cells. The fold increase in the NKG2D gMFI in the total pool of pNK cells incubated with spheroids (**left**) and the fold increase in the NKG2D gMFI in the population of degranulated CD107a^+^ pNK cells (**right**) are shown. Each point on the graph is the result of measuring one individual sample. E:T = 3:1, incubation time = 24 h. Statistical analysis was performed using a Kruskal–Wallis multiple comparison test (for (**C**–**E**)) or a nonparametric Mann–Whitney test (for (**A**,**B**)) (* *p* < 0.05, ** *p* < 0.01, *** *p* < 0.005, **** *p* < 0.0001); the means ± SDs are shown.

Among the pNK cells degranulating in response to spheroids, regardless of tumor cell type, there were lower numbers of CD16^+^ cells and higher numbers of cells expressing the KIR2DL2/DL3 inhibitory receptors than in CD107a-negative cells (Figure 5A,B). This observation is in accordance with the findings of this model of natural cytotoxicity involving the predominant participation of mature, educated NK cells, for which the expression of the CD16 receptor, which provides ADCC, is not necessary.

The Tim-3 coinhibitory receptor is involved in the regulation of NK cell activity [33]. Surface Tim-3 expression was upregulated in pNK cells in response to SKOV-3 and BT-474 tumor spheroids; however, no significant difference in Tim-3 expression was observed after coincubation with MCF-7 spheroids. In degranulating pNK cell subsets, Tim-3 expression was greater than in CD107a-negative cells (Supplementary Figure S4A), and after incubation with MCF7 spheroids, the Tim-3 level in this pNK cell fraction was again lower than that in other types of spheroids (Figure 5C).

NK cells with high expression of the CX3CR1 chemokine receptor were defined as generally more cytotoxic than NK cells showing no or low CX3CR1 expression [34]. In our model, we did not observe an increase in the surface CX3CR1 level in degranulating pNK cell fractions; however, compared with those in SKOV-3-coincubated NK cells, both the total and the fraction of degranulating pNK cells against BT-474 and MCF7 spheroids exhibited significantly lower CX3CR1 surface density (Figure 5D, Supplementary Figure S4B).

Incubation of pNK cells with all three types of tumor spheroids led to a decrease in the surface expression of NKG2D to various degrees, in contrast to that of Tim-3 and CX3CR1 (Figure 5E). Moreover, we detected greater expression of NKG2D among degranulating CD107a^+^ cells than among resting CD107a^−^ NK cells (Supplementary Figure S4C). Compared with those in SKOV-3 spheroids, the surface levels of NKG2D in both total and degranulated pNK cells were increased after coculture with BT-474- or MCF7-derived spheroids (Figure 5E).

### 3.4. Ligands in 3D Cultures Modulate Changes in the Phenotype of pNK Cells

To further explore the involvement of the NKG2D, Tim-3 and CX3CR1 receptors in the NK-cell-mediated cytotoxic effects on tumor spheroids, we measured the levels of several molecules that could serve as ligands for the receptors on the surface of tumor cells after spheroid dissociation.

**Figure 6:**
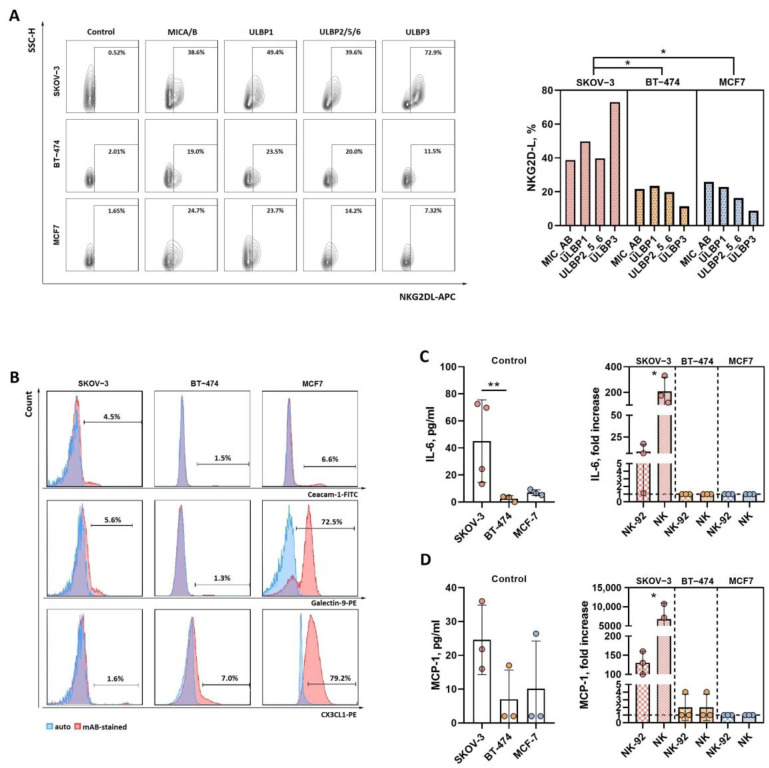
Phenotype and cytokine production of spheroids derived from SKOV-3 and BT-474 и MCF-7 cells. (**A**) Expression analysis of NKG2D ligands (MICA/B, ULBP1, ULBP2/5/6 and ULBP3) in SKOV-3, BT-474 and MCF7 spheroids. Representative flow cytometry data (left) and comparative analysis of NKG2DL expression (right). (**B**) The expression of Tim-3 ligands (CEACAM1 and galectin-9) and the CX3CR1 ligand (CX3CL1) in SKOV-3, BT-474 and MCF7 spheroid cells; representative cytometry data are shown. (**C**,**D**) Multiplex analysis of soluble IL-6 (**C**) and MCP-1 (**D**) in supernatants from SKOV-3, BT-474 and MCF-7 spheroids incubated with or without (control) NK-92 or pNK cells. E:T = 3:1, incubation time = 24 h. Statistical analysis was performed using a nonparametric Mann–Whitney U test (* *p* < 0.05, ** *p* < 0.01); the means ± SDs are shown.

Cell surface NKG2D ligands (NKG2DLs) are known to be potent activators of NK cell cytotoxicity, and their expression varies among cell types [35]. In humans, these ligands include the highly polymorphic MHC class I-related proteins MICA and MICB and UL16-binding proteins (ULBPs). We examined the surface expression of MICA/B, ULBP1, ULBP2/5/6 and ULBP3 on SKOV-3, BT-474 and MCF-7 tumor cells growing in 3D culture after spheroid dissociation. All three types of cells expressed NKG2DL, although to various degrees. The proportions of NKG2DL-positive cells, including MICA/B-, ULBP1-, ULBP2/5/6- and ULBP3-expressing cells, were greater in SKOV-3 cells than in BT-474 and MCF-7 cells (Figure 6A). Notably, in SKOV-3 cells, ULBP3 was the predominant ULBP3 among the ULBPs.

The most studied Tim-3 ligands are galectin-9 and CEA cell adhesion molecule 1 (CEACAM1), which are known to regulate cytokine production and the cytotoxicity of NK cells [36]. We analyzed the surface expression of these molecules in SKOV-3, BT-474 and MCF7 tumor spheroids. BT-474 cells did not express galectin-9 or CEACAM1; SKOV-3 cells were weakly positive for these Tim-3 ligands; and MCF-7 cells were characterized by high expression of galectin-9 (Figure 6B).

We also measured the expression of fractalkine (CX3CL1) on tumor cells of all three types. BT-474 and MCF7 cells showed low (less then 10%) and high (approximately 80%) positivity for CX3CL1, respectively; SKOV-3 cells almost did not express this ligand for CX3CR1 on their surface in a 3D culture.

Additionally, we assessed the secretory activity of tumor-cell spheroids after interaction with NK effector cells. Analysis of the 3D-culture supernatants showed that the secretion of IL-6 was induced in only SKOV-3 cells by both pNK and NK-92 cells (Figure 6C), as was the secretion of monocyte chemoattractant protein-1 (MCP-1) (Figure 6D).

Thus, SKOV-3 cells exhibited the highest expression levels of NKG2D ligands and were induced to secrete soluble factors involved in the regulation of the immune response in the tumor microenvironment. Therefore, the increased susceptibility of SKOV-3 cells to pNK cells observed in our model is at least in part mediated by the contribution of the NKG2D/NKG2DL axis to cell activation and secretory activity. MCF-7 cells overexpressed surface galectin-9 and CX3CL1. Together with the decreases in Tim-3 and CX3CR1 levels in pNK cells after interaction with MCF-7 spheroids, these findings may indicate the involvement of the Tim-3/galectin-9 and CX3CR1/CX3CL1 pathways in the regulation of the cytotoxic response of pNK cells to MCF-7 tumor cells in the 3D model.

## 4. Discussion

NK-cell-based products, both conditioned peripheral NK cells and those based on the NK-92 cell line, are currently being actively tested in cancer therapy. All of these approaches have their own advantages and weaknesses, and their therapeutic effects may differ depending on the type of tumor. The stable NK-92 cell line differs from primary pNK cells by its unlimited proliferative potential, which allows the cells to be successfully accumulated in vitro in the required quantities, while the expansion and activation of peripheral blood NK cells in vitro is still a challenge [5]. In turn, NK cells contain multiple subsets that vary in their differentiation stage, “licensing” and expression of activating and inhibitory receptors, which may improve the cytotoxic response to different tumors. Although the NK-92 cell line possesses strong lytic potency, the significantly greater expression of Eomes in these cells than in peripheral blood CD56^dim^ NK cells allows us to assign NK-92 cells to the CD56^bright^-like phenotype [17]. A direct comparison of the cytotoxic effects of these cells in an experiment can ultimately provide new insight into which of these cellular variants can become the basis for obtaining an optimal therapeutic product.

In this work, by determination of the viability of tumor-cell spheroids growing in one-size molds via optimized flow-cytometry-based analysis, we explored the differences in susceptibility of certain tumor cells to the cytotoxic effects of NK-92 cells and preactivated pNK cells. Among the reasons for the variability may be specific tumor cell characteristics that increase susceptibility to one or more NK cell subsets. A comparison of the SKOV-3, BT-474 and MCF7 spheroids revealed their various growth dynamics and sizes. While BT-474 and MCF7 spheroids enlarged during cultivation for 6 days, the size of the SKOV-3-derived spheroids slightly decreased over this time period (Supplementary Figure S1). A tendency to shrink during spheroid formation was observed for some cell lines and was associated with increased intercellular contact [37]. Controlling the characteristics of the spheroids, especially the uniformity of shape and size, enhances the reproducibility of the analyzed results [38]. The 3D model used in our work allows the production of multiple individual tumor spheroids of similar size by using identical agarose forms and provides the possibility of analyzing the effects of NK cells under comparable conditions.

We detected the lowest NK-92-cell-mediated cytotoxic effect, among the three tumor cell lines tested, against MCF7 spheroids, whereas the BT-474 spheroids were obviously most resistant to the pNK cells. Interestingly, the MCF7 spheroids were more susceptible to pNK cell action than were the NK-92 cells. The most pronounced cytotoxic effects of both NK-92 and pNK effector cells, which had the highest percentage of dead cells and an increased proportion of degranulated pNK cells, were observed in SKOV-3 3D cultures. Further use of fluorescently labeled SKOV-3 (SKOV-Kat) spheroids and CLSM analysis of live SKOV-Kat cells revealed that the cytotoxic activity of the pNK cells against the spheroids was superior to that of the NK-92 cell line.

Among the factors that determine the success or failure of NK cell activity are the surface molecules expressed by both the target and effector cells and the cytokines produced by the interacting cells. In addition, the production of certain cytokines may be considered a sign of NK cell activation. In our models, the secretion of IFNγ, TNF and GM-CSF by pNK cells predominated over the cytokine production by NK-92 cells and was upregulated in response to SKOV-3 spheroids, whereas the interaction of NK-92 or pNK cells with BT-474 and MCF7 spheroids did not induce an increase in the production of these cytokines.

Different susceptibilities of tumor-cell spheroids to soluble NK-cell-derived factors likely contributed to the increase in tumor cell death in our models. The production of IFNγ is not only a major factor in the antiviral and bacterial activity of NK cells but also a factor that may promote the apoptosis and cytolysis of target tumor cells [39]. However, some studies have shown that IFNγ in the tumor microenvironment (TME) may promote cancer immunosurveillance in the presence of NK cells [40]. TNF caused apoptosis in MCF7 cells [41], while HER2-positive cell lines, such as SKOV-3 and BT-474, were shown to be resistant to TNF [42]. Moreover, TNF contributes to the proinflammatory microenvironment and favors the activation and degranulation of mature cytotoxic NK cells [43].

The increase in GM-CSF production by pNK cells observed in the SKOV-3-based 3D model indicates the activation of these effector cells. However, the role of this cytokine in cancer is ambiguous. Although some studies have shown that GM-CSF has an inhibitory effect on tumor growth and metastasis, many additional studies have shown that GM-CSF may have a stimulating effect on tumor progression [44]. SKOV-3-induced GM-CSF secretion by NK cells may be associated with the decreased ability of these tumor spheroids to express TGF-β compared to that of BT-474 and MCF-7 cells (Supplementary Figure S5). Previously, TGF-β was shown to suppress GM-CSF production in NK cells [45]. A high density of intercellular contacts, heterogeneity, hypoxia and TGF-β overexpression in solid tumors contribute to the downregulation of activating NK cell receptors [46]. Apparently, primary NK cells, but not the NK-92 cells, are programmed to produce GM-CSF after interacting with tumor cells for the recruitment of other immune cells in vivo.

The mechanisms of natural cytotoxicity are supposed to largely determine the difference in susceptibility to NK cell attack among tumor spheroids. Differential gene expression defines a variety of mechanisms of NK cell cytotoxicity depending on the cancer cell line [47]. Many NK cell surface molecules drive cytotoxic reactions and cytokine production upon ligand recognition. The molecule pool includes activating and inhibitory NK cell receptors that interact with classical and nonclassical MHC class I molecules and stress-inducing ligands. To determine the specific effector–target interactions underlying the variation in the cytotoxic effects of pNK cells on tumor cells, we analyzed some phenotypic features of total and degranulating NK cells after coculture with tumor spheroids. Simultaneous comparison of the three tumor lines allowed us to level out variations in effect associated with differences in the NK cells from different donors.

The phenotypic heterogeneity of pNK cells is associated with variations in the functional repertoire. Mature NK cells are considered to be active cytotoxic effectors, while less differentiated subsets mostly play a regulatory role and boost other immune cells via cytokines. In addition to the typical CD56, CD57 and NKG2A marker distributions that mostly characterize the maturation grade, a wide spectrum of differentially expressed genes helps to define three subtypes (clusters) of NK cells that demonstrate distinct functional characteristics [32]. NK cells poorly infiltrate solid tumors, and the phenotype of the infiltrating fraction depends on the tumor type. Compared with those in peripheral blood, NK cells in the NK2 cluster are enlarged in breast and ovarian cancer tissues; however, these cells often exhibit features of a dysfunctional state [48]. NK1 cells are less susceptible to the suppressive TME and may exhibit intrinsic antitumor cytolytic activity. The NK1 cluster is characterized by high expression of CD16, CX3CR1, inhibitory KIRs and Tim-3 receptors, which take part in the recognition of ligands expressed by cancer cells that regulate cytotoxic reactions. NK1 cells constitute more than half of the total NK cells found in tumors in breast and ovarian cancers [32,48]. The NK2 cluster was defined by little or no expression of CD16 or CD57 and high expression levels of CD56 and NKG2A, along with NKG2D overexpression, indicating that the NK2 subset comprised CD56^bright^ and early-stage CD56^dim^ NK cells. The ratio of NK cells with a CD56^bright^ dysfunctional phenotype to those with a CD56^dim^ cytotoxic effector phenotype can be a prognostic factor in cancer, and the results of our study of the NK cell effector phenotype could be used to determine the most effective therapeutic strategy.

Assessment of NK cell degranulation in response to SKOV-3, BT-474 and MCF7 spheroids revealed NK cell fractions with enhanced effector activity against these target cells. SKOV-3- and MCF-7-derived spheroids produced greater numbers of CD107a^+^ NK cells than did BT-474 spheroids (Figure 4), which corresponds well with the tumor cell viability data obtained in the presence of NK cells (Figure 1). When characterizing the phenotype of pNK effector cells, we first focused on markers of the NK1 cluster, such as inhibitory KIRs, CD16, Tim-3 and CX3CR1. We showed that the proportion of KIR2DL2/3^+^ cells increased among degranulating pNK cells. These cells usually represent a fraction of mature, educated NK cells capable of natural cytotoxicity [49]. KIR expression was shown to be prominent in the NK1 cluster [32]. Notably, we have previously shown that KIR-positive cells are more susceptible to retroviral transduction; thus, it is possible to create highly toxic effectors for cancer cell therapy [50].

According to our model, the level of Tim-3 increased on pNK cells after interaction with SKOV-3 and BT-474 spheroids, which clearly indicated NK cell activation, while no such increase was recorded with MCF7 spheroids. It is possible that the internalization of Tim-3 induced by the receptor recognition of galectin-9, which is highly expressed by MCF-7 spheroids, may be a reason for the decreased Tim-3 level in pNK cells after interaction with MCF-7 cells compared to that in other tumor cell spheroids. We also detected an increase in Tim-3 in the degranulating NK cell fraction. This finding is consistent with previous observations that Tim-3 expression marks effector NK cells that undergo degranulation [51]. It was also shown that activation of the Tim-3/galectin-9 pathway can promote a significant increase in IFNγ production [52]. Unlike T cells, in which Tim-3 expression is considered a strong marker of functional exhaustion, Tim-3 expression in NK cells is not always associated with the exhaustion state and is regulated by various factors, including the mode of activation [51,52,53]. On the other hand, several works support the involvement of Tim-3 in the negative regulation of the NK cell immune response. Interactions of Tim-3 with the membrane-bound or soluble CEACAM1 and/or galectin-1 produced by tumor cells led to the inhibition of NK-cell-mediated tumor cell killing independent of MHC class I status [54]. The role of Tim-3 in regulating NK cell activity is incompletely understood; possibly, the effect of Tim-3 might be activating or inhibitory, depending on the type of ligand.

Interestingly, in the pool of degranulating pNK cells, the samples containing pNKs and target cells clustered according to pNK receptor expression, and these clusters corresponded to a certain kind of target spheroid (Supplementary Figure S6). NK cells that degranulated during coincubation with SKOV-3 and BT-474 cells were characterized by a high expression level of Tim-3 and formed cluster 2, while NK cells that responded to MCF7 spheroids with a large fraction of galectin-9^+^ cells had a low expression level of Tim-3 and formed cluster 1 (Supplementary Figure S6A).

Another receptor associated with the cytotoxic NK1 cluster is CX3CR1. We have shown the different effects of different tumor spheroids on the surface level of CX3CR1 in pNK cells. Specifically, CX3CR1 levels were greater in effector pNK cells after coculture with SKOV-3 target cells than after coincubation with BT-474 or MCF7 spheroids. In addition, compared with those of SKOV-3 spheroids, the fractions of pNK cells that degranulated in response to BT-474 and MCF7 cells had significantly lower expression levels of CX3CR1. These effects might also be associated with receptor internalization upon ligand recognition [55], as long as both the BT-474 and MCF7 spheroids contained a CX3CL1-positive fraction, whereas the SKOV-3 spheroids did not express CX3CL1. Moreover, we detected no differences in the CX3CR1 surface density between degranulated and nondegranulated pNK cell fractions, regardless of the type of tumor cells with which the pNK cells were incubated. Therefore, the CX3CR1-CX3CL1 axis apparently plays no role in the degranulation process, and CX3CR1 expression may reveal functionally active NK cell subsets and indicate that CX3CR1-positive cells are more cytotoxic than are other cells [56]. Additionally, CX3CR1 is considered an additional differentiation marker that may link NK cell maturation to the ability to migrate [34].

Even though the proportions of CD107a^+^ cells in the presence of SKOV-3- and MCF7-derived spheroids were almost identical, pNK cells incubated with SKOV-3 cells were characterized by greater cytokine production than were those incubated with MCF7 spheroids. This difference was especially noticeable in the pool of degranulating pNK cells after incubation with SKOV-3 spheroids. According to a recent report, the NK1 subset contains a fraction of cells that are able to express granzyme B and perforin but also tend to produce more cytokines, such as IFNγ and TNF, than other clusters. These cells, similar to the less cytotoxic NK2 cells, are characterized by high expression of NKG2D, one of the main cytotoxicity-associated receptors of NK cells [32].

In our model, the SKOV-3 spheroids that were the most sensitive to the cytotoxic impact of NK cells were characterized by the highest expression levels of NKG2D ligands, such as MICA/B and ULBPs. For all three types of spheroids, increased NKG2D levels were observed in the pool of degranulating CD107a^+^ pNK cells, which supports the active involvement of NKG2D-expressing pNK cells in the cytotoxic reaction against these tumor cells. Moreover, NKG2D downmodulation was observed as a result of pNK cell interactions with tumor spheroids; this effect was greater when pNK cells were incubated with SKOV-3-derived spheroids than when they were incubated with BT-474 or MCF-7 spheroids. We again detected well-defined clusters of degranulated NK cells depending on the expression of NKG2D and CX3CR1 on the cell surface. The NK cells that reacted with the NKG2DL^+^ CX3CL1^−^ SKOV-3 spheroids were characterized by the NKG2D^low^CX3CR1^high^ phenotype (cluster 3), while the NK cells that reacted with the BT-474 and MCF7 cells, which were characterized by a low proportion of NKG2DL cells and a fraction of CX3CL1^+^ cells, led to the degranulation of NK cells with the NKG2D^high^CX3CR1^low^ phenotype (cluster 4) (Supplementary Figure S6B). Notably, a decrease in the NKG2D level is not always associated with a decrease in NK cell cytotoxic functions, as other activating receptors can be triggered. There is some evidence to suggest that internalization of NKG2D is required for correct activation of intracellular pathways that are crucial for successful NK cell function [57,58,59]. Some in vivo experimental data have been reported recently supporting our findings regarding the eminent role of some NK cell receptors in the tumor elimination process. For instance, the NKG2D/NKG2DL axis in animal models of cancers highlights the complex relationship between the NKG2D expression and NKG2D-ligand-expressing cancer cells for cancer development [60]. The cytotoxic efficacy of modified NKG2D-CAR-NK cells against various types of malignancies, including hematological diseases [61] and solid cancers such as lung [62] and colorectal cancer [63], has been confirmed. An antitumor response driven by the CX3CL1-CX3CR1 axis was evaluated in vivo in a CX3CR1-deficient murine model of melanoma [64] and lung carcinoma [65]. The results of these studies supported the significant role of NK cell activity in antitumor effects against CX3CL1-positive cancers. In a tumor-bearing mouse model, the association of Tim-3 levels in NK cells with tumor growth was shown [66].

The antitumor response of NK cells is affected to different degrees by immunoactive factors secreted by tumor cells and cells in the microenvironment. An increase in the IL-6 level in culture supernatants in response to NK-92 and, to a greater extent, to pNK cell coincubation was exclusively associated with SKOV-3-derived spheroids. Human NK cells exhibit low expression or absence of the IL-6 receptor alpha chain [67]; however, the regulation of ovarian carcinoma SKOV-3 cell proliferation and secretion by autocrine IL-6 has been described [68]. We observed a similar trend for MCP-1 (CCL2) secretion by SKOV-3-derived spheroids. The CCL2/CCR2 axis plays multiple protumorigenic roles and is implicated in cancer formation and metastasis, tumor cell invasion and the promotion of angiogenesis [69]. Moreover, NK cells can induce the expression of the MCP-1 receptor CCR2 [70]. Thus, the CCL2/CCR2 signaling pathway is obviously involved in the interactions between NK and SKOV-3 cells.

## 5. Conclusions

In this work, we showed that NK-92 cells and peripheral blood NK cells possess different antitumor activities against 3D tumor cultures that were determined by the different receptor–ligand axes mediating tumor cell recognition and subsequent elimination. A subset of highly cytotoxic cytokine-producing pNK cells was involved in SKOV-3 spheroid recognition through the NKG2D-NKG2DL pathway, while MCF7 spheroids did not induce cytokine production by pNK cells and showed involvement of the CX3CR1-CX3CL1 axis in degranulation.

Thus, for certain types of cancer, NK-92 cells may have inferior antitumor activity than pNK cells, and the optimal type of effector cell can be selected during preliminary testing of tumor cell susceptibility, taking into account the involvement of certain receptor–ligand axes. Methods, including enrichment of NK cell subsets with high cytotoxicity potential, as described in this work, or genetic modification of effectors to target them to the tumor, taking into account its phenotype, can be further considered to determine the optimal strategy for NK-cell-based antitumor immunotherapy.

## Data Availability

The datasets generated and analyzed during the current study are available from the corresponding author on reasonable request.

## Supplementary Materials

The following supporting information can be downloaded at: https://www.mdpi.com/article/10.3390/biomedicines12102398/s1, Figure S1: Growth kinetics of spheroids derived from BT-474, MCF-7 and SKOV-3 cells after 2, 4 and 6 days. (**A**–**C**) Stereomicroscopic imaging of the BT-474, MCF7 and SKOV-3 spheroids. The scale bar corresponds to 1000 µm. (**D**–**F**) Area and diameter of the spheroids. The values were averaged over 6-7 individual BT-474, MCF7 and SKOV-3 spheroids. Statistical analysis was performed using a Kruskal–Wallis test (* *p* < 0.05, ** *p* < 0.01, *** *p* < 0.005); the means ± SDs are shown. Figure S2: Assessment of the viability of tumor cells extracted from spheroid SKOV-3. (**A**,**B**) Comparative analysis of the proportion of SytoxBlue^+^ cells among intact SKOV-3 or SKOV-Kat spheroids after incubation with pNK cells. (**C**) Correlation analysis between the number of live (Katushka^+^) cells and the number of dead (SytoxBlue^+^) cells. Figure S3: Soluble GrnB in equal amounts of NK-92 and pNK cells (**left**) and the fold change in soluble GrnB after mixing with SKOV-3, BT-474 and MCF-7 spheroids (**right**). Figure S4: Analysis of markers characterizing activation and differentiation stages among CD107a^+^ and CD107a^−^ NK cells in response to tumor spheroids consisting of BT-474, MCF-7 or SKOV-3 cells. (**A**) Comparison of the fold change in Tim-3 gMFI between degranulated (CD107a^+^) and resting (CD107a^−^) pNK cells. (**B**) Comparison of the fold change in gMFI between degranulated (CD107a^+^) and resting (CD107a^−^) pNK cells. (**C**) Comparison of the fold change in NKG2D gMFI between degranulated (CD107a^+^) and resting (CD107a^−^) pNK cells. Statistical analysis was performed using a nonparametric Mann–Whitney test (* *p* < 0.05, ** *p* < 0.01); the means ± SDs are shown. Figure S5: TGF-β expression levels in SKOV-3, BT-474 and MCF7 tumor spheroids. Three-dimensional samples were pooled from 2 molds, total RNA was isolated with ExtractRNA reagent and cDNA was synthesized using a qPCRmix-HS SYBR kit (all from Evrogen, Russia). The hypoxanthine-guanine phosphoribosyltransferase (HPRT1) and β-actin housekeeping genes were used as reference genes for normalization. Amplification was performed on a CFX96 real-time system (Bio-Rad). The data were calculated from three different experiments. The relative quantification of the mRNA levels of the target genes was performed using CFX Manager software 3.1 based on the threshold cycle method. The results reflect the PCR signal for the target transcript normalized to 2D conditions, arbitrarily set to 1. Synthetic oligonucleotides: TGFb (for: acctgaacccgtgttgctc; rev: ctaaggcgaaagccctcaat); actin (for: tcctgtggcatccacgaaact; rev: gaagcatttgcggtggacgat); HPRT1 (for: cctgctggattacatcaaagcactg; rev: tccaacacttcgtggggtcct). The data are shown as the mean ± SD. Figure S6: Correlation between the expression levels of receptors and the fraction of degranulating CD107a^+^ pNK cells. (**A**) Correlation between the expression levels of the receptors Tim-3 and CX3CR1 in the pool of degranulating CD107a+ pNK cells. (**B**) Correlation between the expression levels of the receptors CX3CR1 and NKG2D in the pool of degranulating CD107a^+^ pNK cells. Statistical analysis was performed using Spearman’s correlation test (* *p* < 0.05). Table S1: Demographic characteristics of donors who participated in the study.
