# Supplementary material for: Three-Dimensional Model Analysis Revealed Differential Cytotoxic Effects of the NK-92 Cell Line and Primary NK Cells on Breast and Ovarian Carcinoma Cell Lines Mediated by Variations in Receptor–Ligand Interactions and Soluble Factor Profiles
Source: Biomedicines. 2024 Oct 20;12(10):2398. doi: 10.3390/biomedicines12102398 (PMC11504426; doi:10.3390/biomedicines12102398)
Supplement: Supplementary file 1 [file biomedicines-12-02398-s001.zip › biomedicines-3258827-supplementary/Supplementary Table S1.pdf]

Supplementary Table S1. Demographic characteristics of donors participated in the study.

Number of donor	Age	Gender
1	29	Male
2	30	Female
3	59	Female
4	41	Female
5	30	Male
6	26	Female
7	32	Male
8	30	Male
9	31	Male
