# Supplementary figures and images for: Three-Dimensional Model Analysis Revealed Differential Cytotoxic Effects of the NK-92 Cell Line and Primary NK Cells on Breast and Ovarian Carcinoma Cell Lines Mediated by Variations in Receptor–Ligand Interactions and Soluble Factor Profiles
Source: Biomedicines. 2024 Oct 20;12(10):2398. doi: 10.3390/biomedicines12102398 (PMC11504426; doi:10.3390/biomedicines12102398)

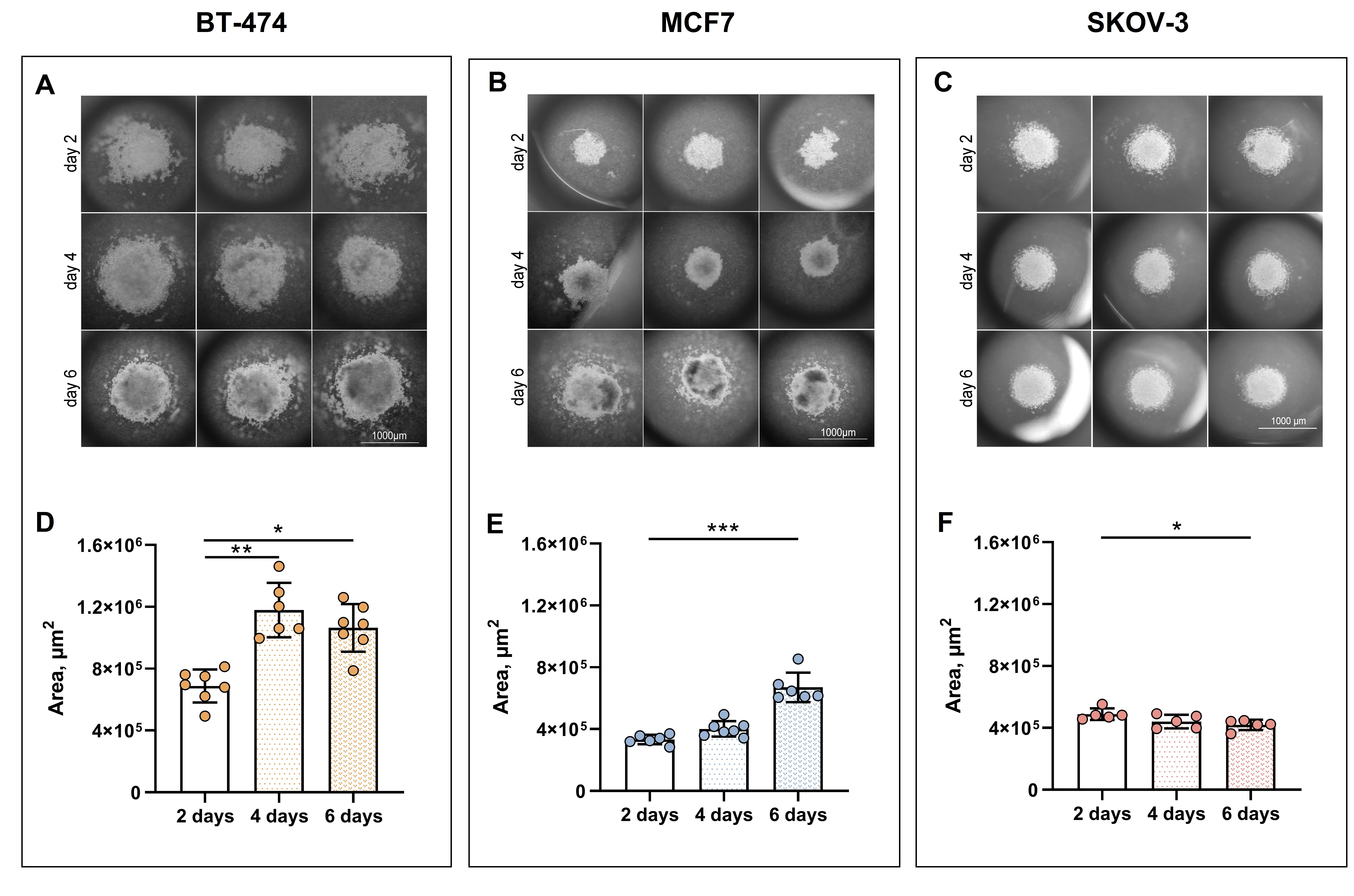

Supplement: Supplementary file 1 [file biomedicines-12-02398-s001.zip › biomedicines-3258827-supplementary/Supplementary Figure S1.tif]

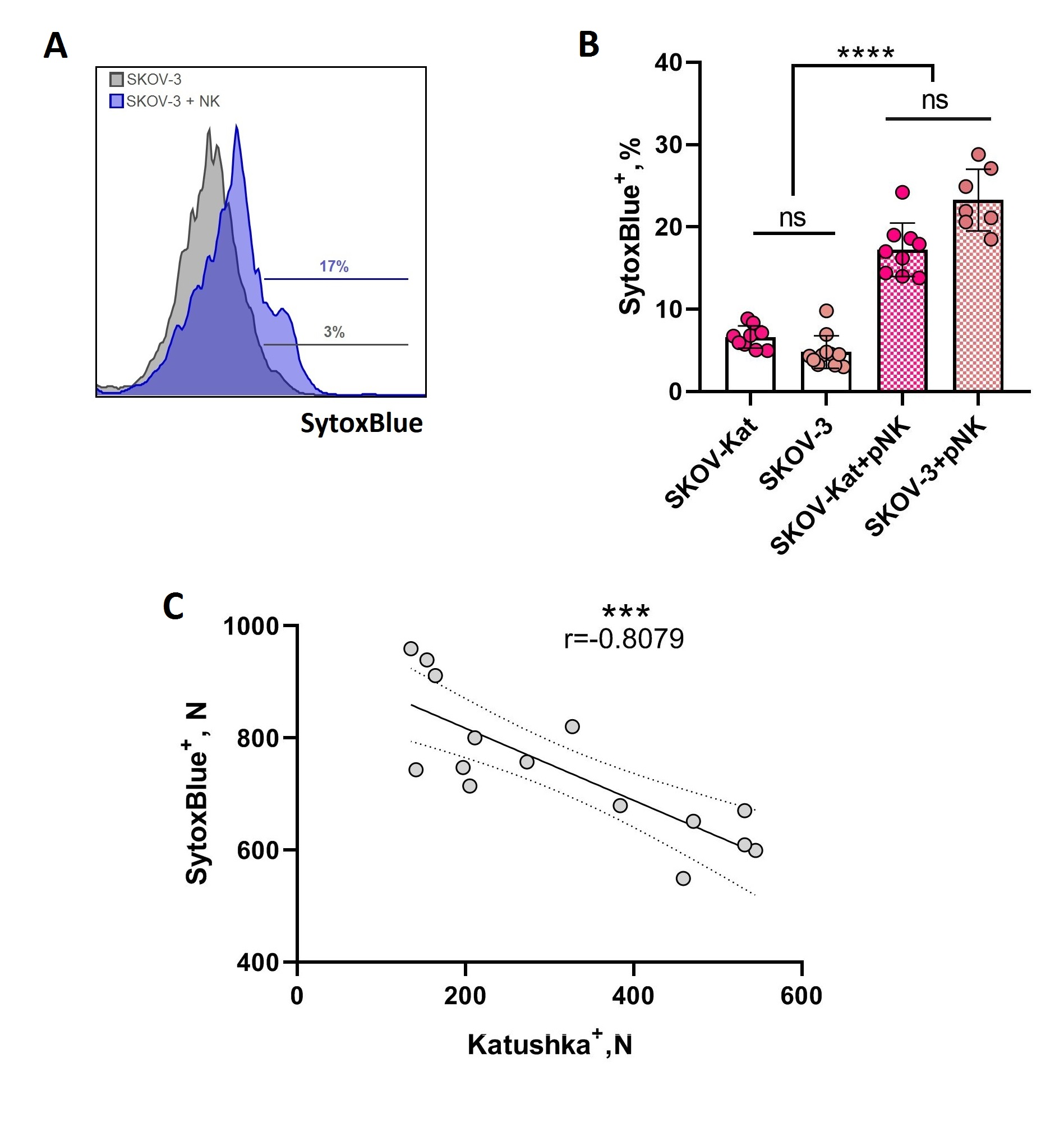

Supplement: Supplementary file 1 [file biomedicines-12-02398-s001.zip › biomedicines-3258827-supplementary/Supplementary Figure S2.tif]

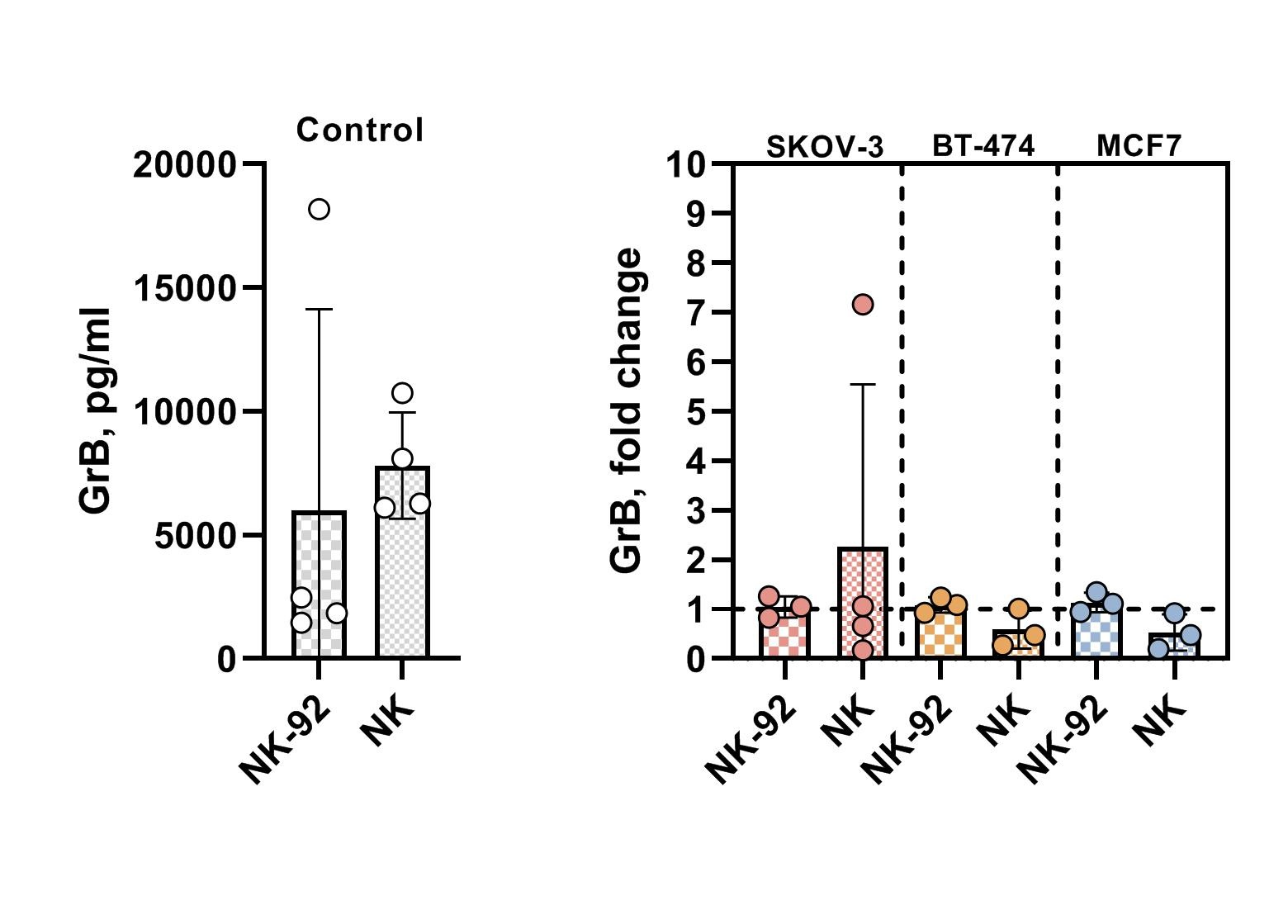

Supplement: Supplementary file 1 [file biomedicines-12-02398-s001.zip › biomedicines-3258827-supplementary/Supplementary Figure S3.tif]

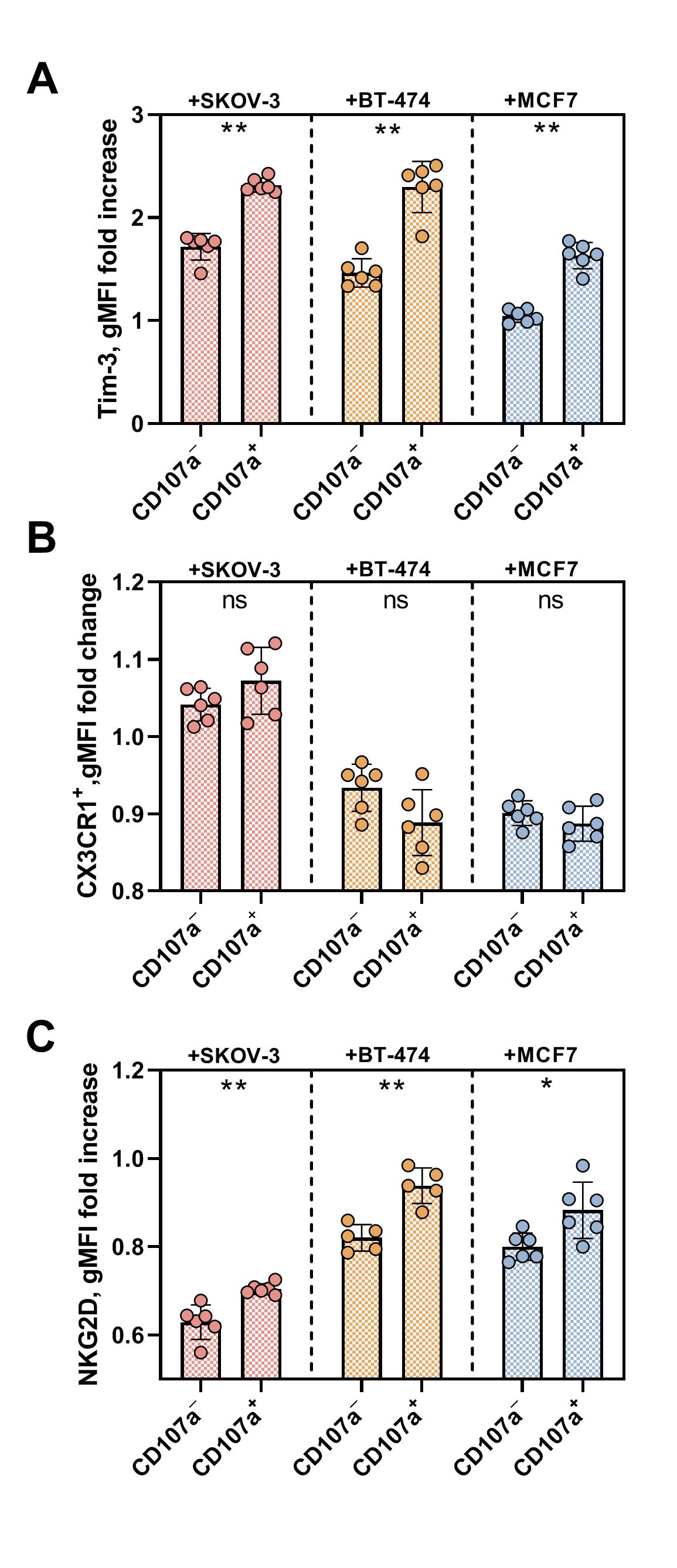

Supplement: Supplementary file 1 [file biomedicines-12-02398-s001.zip › biomedicines-3258827-supplementary/Supplementary Figure S4.tif]

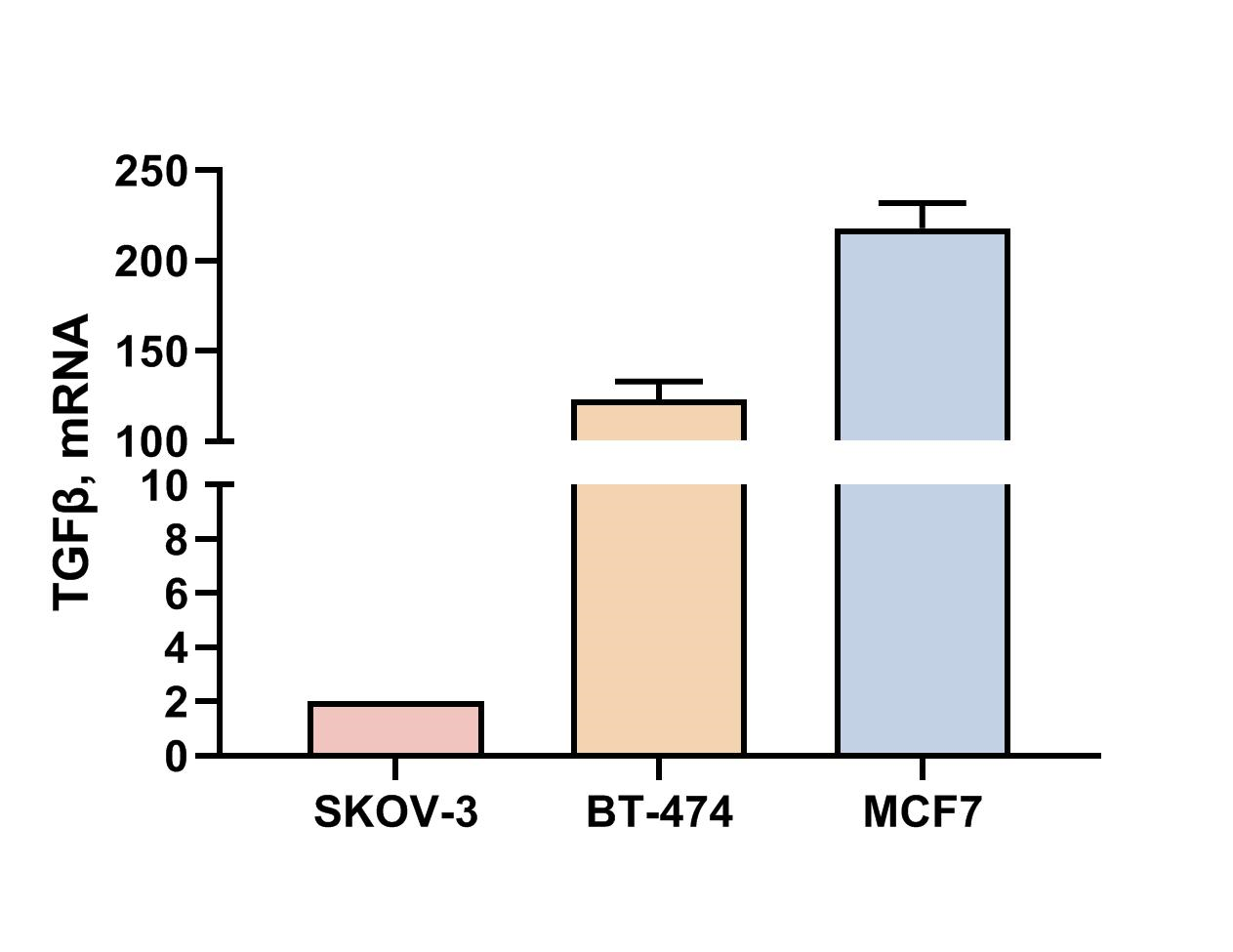

Supplement: Supplementary file 1 [file biomedicines-12-02398-s001.zip › biomedicines-3258827-supplementary/Supplementary Figure S5.tif]

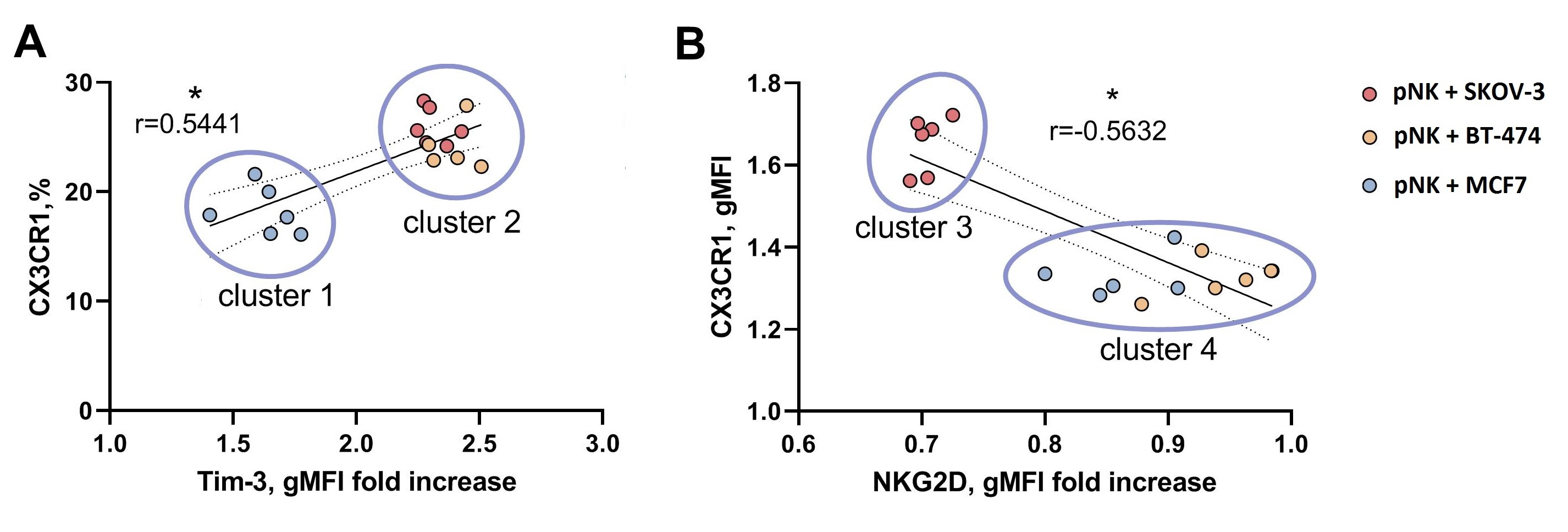

Supplement: Supplementary file 1 [file biomedicines-12-02398-s001.zip › biomedicines-3258827-supplementary/Supplementary Figure S6.tif]
